# Fatal Stroke after the Death of a Sibling: A Nationwide Follow-Up Study from Sweden

**DOI:** 10.1371/journal.pone.0056994

**Published:** 2013-02-22

**Authors:** Mikael Rostila, Jan Saarela, Ichiro Kawachi

**Affiliations:** 1 Centre for Health Equity Studies, Stockholm University/Karolinska Institutet, Stockholm, Sweden; 2 Åbo Akademi University and University of Helsinki, Vasa and Helsinki, Finland; 3 Harvard School of Public Health, Boston, Massachusetts, United States of America; University of Queensland, Australia

## Abstract

**Background:**

Although less studied than other types of familial losses, the loss of a sibling could be a potential trigger of stroke as it represents a stressful life event. We studied the association between loss of a sibling and fatal stroke up to 18 years since bereavement**.**

**Methodology/Principal Findings:**

We conducted a follow-up study between 1981 and 2002, based on register data covering the total population of Swedes aged 40–69 years (n = 1,617,010). An increased risk of fatal stroke (1.31 CI: 1.05, 1.62) was found among women who had experienced the loss of a sibling. No increase in the overall mortality risk was found in men (1.11 CI: 0.92, 1.33). An elevated risk in the short term (during the second and third half-year after the death) was found among both men and women, whereas longer-term elevation in risk was found primarily for women. Both external (1.47 CI: 1.00, 2.17) and not external (1.26 CI: 1.00, 1.60) causes of sibling death showed associations among women. In men, an association was found only if the sibling also died from stroke (1.78 CI: 1.00, 3.17). However, among women, we found an increased risk of stroke mortality if the sibling died from causes other than stroke (1.30 CI: 1.04, 1.62).

**Conclusions/Significance:**

The findings suggest an increased risk of dying from stroke mortality after the death of a sibling, and that bereavement affects particularly women. It is important for health care workers to follow bereaved siblings and recognize potential changes of stress-levels and health related behaviours that could lead to risk of stroke.

## Introduction

The loss of a family member is known to influence health and mortality among surviving family members [1]–[8]. Yet bereavement and mortality after a sibling’s death has been rarely studied [9]–[11], although it is a common event. In the U.S., for instance, almost 2 million minor children lose a sibling each year [12]. To the extent that siblings are also beloved, provide companionship and support, one would expect that death of an adult sibling – as much as the death of other family members (e.g. spouse, parents, children) – could be considered a stressful life event and a potential trigger of stroke. In fact, the death of a sibling often represents the loss of the longest and most intimate relationships of a person’s lifetime [13]. Some studies even suggest that the death of a sibling is more disruptive and involves a more severe grief process than other familial deaths [11]. Accordingly, it has been shown that associations between sibling deaths and mortality among remaining siblings are comparable and, in some instances, stronger than other familial deaths [11], [14] including the loss of a child [15]. Moreover, the fact that findings on the duration of grief following familial deaths are unclear [16] stresses the importance of studying whether associations are discerned in shorter or longer-term perspectives.

Several studies suggest that the risk of fatal and non-fatal stroke is influenced by stress and stressful life events [17]–[19]. Losing a sibling represents such a stressful life event, and could trigger stroke through acute psycho-physiological stress mechanisms [1], [2]. Deaths from such acute stress mechanism could appear within the first few hours and days after a stressful event. Chronic stressors following bereavement could, however, also lead to pathophysiological changes in the sympathetic nervous system, the hypothalamic-pituitary-adrenalaxis, and the immune system [1], [2]. Pathophysiological changes could lead to stroke months or years after bereavement and primarily lead to an increased risk if the death is unexpected and hence more stressful [16]. In addition, deleterious coping responses, such as smoking, increased alcohol consumption, and poor diet and exercise habits could also follow bereavement [2], [3]. Studies have shown that such behaviours contribute to the risk of stroke [20]–[24] and they are therefore likely to contribute to increased risk of stroke over the longer term. Although there are strong reasons to believe that sibling deaths and the stress and grief that it involves could have an impact on surviving siblings’ health through the aforementioned mechanisms it should be emphasized that knowledge on active mechanisms is still scarce.

There are reasons to expect sex differences in the association between sibling death and subsequent health of the surviving sibling. A review suggests that men in particular, appear more vulnerable during the earlier months of bereavement after the loss of a spouse while the risk period is longer for women [25], which might be due to the influence of longer-term mechanisms. It has also been found that middle-aged women are especially vulnerable to increased acute emotional stress levels that could follow grief (“the broken heart syndrome”) [26], [27]. However, another study suggests no clear sex differences depending on the time since the death of a sibling [6]. Hence, whether there are sex differences in the association between the loss of a sibling and stroke mortality depending on the time interval since the death remains unclear.

Siblings share a similar biological predisposition to death and disease, which makes confounding by genetic inheritance likely. Siblings also share many environmental exposures during childhood and adolescence. An increased risk of mortality from stroke after a sibling had died from stroke might therefore be a partial marker of shared genetic predisposition or shared environmental determinants to the disease. Accordingly, a recent study suggests a 60 per cent increased risk of stroke if having a sibling with prior stroke [28]. A method of getting closer to causal inference, and hence assist in teasing out causation from confounding, is to examine whether pairs of siblings died of discordant causes or the same specific cause (e.g. stroke).

Our aim was to conduct a large-scale longitudinal study on stroke mortality following the loss of a sibling at adult age, using intergenerational linked data from nationwide Swedish registers. We postulated that the association between sibling’s death and fatal stroke events at adult ages will depend on the time interval since the sibling’s death, the sex of the remaining sibling, as well as the specific cause of death.

## Methods

### Ethics Statement

The data material was approved by the Regional Ethical Review board of Karolinska Institutet in 2002-11-11 (decision no. 02–481) and the Central Ethical Review Board 2012-09-13 (application no. 2012/1260-31). These decisions mean that the data can be used for several purposes. Swedish law allows for using information from registers for research purposes as long as researchers follow the article that deals with the ethics of research that involves humans (Etikprövningslagen, 2003∶460), and they have ethical approval. This regulation exempts our study from requiring written informed consent. All data used here was also anonymous and researchers did not have access to any personal information that could identify study participants (e.g. personal identity number, home address etc.). Consequently, it was not possible to trace specific individuals included in the data material.

### Participants and Procedure

The data come from multiple-linked data of national Swedish routine registers, maintained at the Centre for Health Equity Studies (CHESS) in Stockholm. In the study, all persons born in Sweden during the period 1932–1962 and alive at end-1980 were linked to the mother, provided that she was born in Sweden and alive at end-1980. Hence sibling groups are identified through the mother. Singletons were excluded from analysis. We restrict the data to people aged at least 40 years, since very few persons die from stroke at young adulthood. Under study are consequently persons aged 40–69 years, who can be observed during the period 1981–2002. We were refrained from separating full-siblings and half-siblings, because we then would have to use information about alive fathers. That approach would reduce, and plausibly also askew, the study population. The number of half-siblings would further be too small to facilitate any rigorous analyses concerned with stroke mortality (which also would be the case for any approach to explicitly study twins).

We included individual-level information about basic socio-demographic variables (age, socioeconomic status, marital status, number of children, number of siblings, region of residence, and calendar year) to proxy regional and social differences in stroke mortality as well as the month and cause of death for all persons who died during the study period. Socioeconomic status distinguished blue-collar workers, white-collar workers, self-employed, and people outside the labour market. Marital status consisted of the categories married, previously married, and never married. Number of children and number of siblings were treated as categorical variables. Region of residence refers to each person’s county of residence and consisted of 26 different categories. All covariates except age and calendar year were measured at the end of 1980, which was the only point in time when adequate and complete information was available on them. This was before any sibling death had occurred, and since all people were aged at least 40 years at this time, the variables should proxy the situation at prime working age reasonably well. We distinguished deaths from stroke (cerebrovascular disease), which have the ICD 8 and ICD 9 codes 430–438 and ICD 10 codes I60–I69, and external causes of deaths (predominantly accidents and suicides), which have the ICD 8 codes E800–E999, ICD 9 codes E807–E999, and ICD 10 codes V01-Y98.

All people who experienced a sibling’s death were included, whereas those who did not experience a sibling’s death comprised a ten per cent random sample. In the statistical analyses, people from each group were weighted according to their sampling proportion using normalized weights to correct for inflated t-statistics. The death of sibling is a time-varying feature, which means that when someone’s sibling died, the surviving person changes status from not having experienced to having experienced a sibling’s death. We estimated standardized risks of fatal stroke using Cox regressions, where the focus was on the risk ratio of people who had experienced the death of a sibling and people had not experienced the death of a sibling. The risk was measured at each quarter of a year. Separate analyses were consistently conducted for men and women.

## Results

In total, 65,802 men and 65,118 women experienced a sibling’s death, and 163 and 121 of them subsequently died from stroke, respectively (Table 1). Corresponding numbers in the group who did not experience the death of a sibling were 1,848 deaths in 750,390 men, and 1,186 deaths in 735,700 women. The unadjusted death rate from stroke in persons who had experienced a sibling’s death was approximately twice that of persons who had not experienced a sibling’s death (0.29/0.16 in men, and 0.22/0.11 in women).

**Table 1:** Characteristics of persons with and without sibling loss.

	Men											Women											
	Sibling loss						No sibling loss					Sibling loss						No sibling loss					
	%		Deaths		Rate		%		Deaths		Rate	%		Deaths		Rate		%		Deaths		Rate	
Age in years																							
40–44	16.6		7		0.08		31.5		199		0.06	16.2		7		0.08		31.1		167		0.05	
45–49	22.4		16		0.13		28.8		315		0.10	21.9		14		0.12		28.6		222		0.07	
50–54	25.0		27		0.20		21.1		396		0.16	24.8		29		0.21		21.3		292		0.12	
55–59	20.7		31		0.27		12.1		440		0.32	20.9		29		0.25		12.4		234		0.17	
60–64	11.9		51		0.77		5.1		355		0.61	12.4		28		0.41		5.4		180		0.30	
65–69	3.4		31		1.65		1.2		143		1.07	3.8		14		0.68		1.3		91		0.64	
Socioeconomic status																							
Blue-collarworker	48.0		76		0.29		40.8		769		0.17	36.0		41		0.21		29.7		409		0.12	
White-collarworker	31.2		34		0.20		40.2		540		0.12	32.0		29		0.17		39.8		380		0.09	
Self-employed	10.6		18		0.31		10.8		206		0.17	4.3		8		0.34		4.3		52		0.11	
Outside labourmarket	10.2		35		0.62		8.2		333		0.36	27.8		43		0.28		26.1		345		0.12	
Marital status																							
Married	57.4		81		0.26		60.8		998		0.14	65.8		62		0.17		68.3		745		0.10	
Previouslymarried	8.4		37		0.80		7.5		252		0.29	12.0		21		0.32		10.6		208		0.18	
Never married	34.3		45		0.24		31.7		598		0.17	22.2		38		0.31		21.2		233		0.10	
Number of children																							
0	28.9		49		0.31		29.6		551		0.16	17.3		24		0.25		18.9		228		0.11	
1	19.2		21		0.20		18.8		344		0.16	19.2		24		0.23		19.0		233		0.11	
2	33.3		53		0.29		34.5		540		0.14	38.8		35		0.16		40.4		422		0.09	
>2	18.6		40		0.39		17.1		413		0.21	24.6		38		0.28		21.7		303		0.13	
Number of siblings																							
1	17.7		31		0.32		41.3		727		0.15	18.0		19		0.19		41.7		445		0.10	
2	25.6		35		0.25		29.3		529		0.16	25.1		25		0.18		29.0		367		0.11	
>2	56.8		97		0.31		29.4		592		0.18	56.8		77		0.25		29.3		374		0.11	
Total	100.0		163		0.29		100.0		1,848		0.16	100.0		121		0.22		100.0		1,186		0.11	
Number of person years	552,886						11,383,335					548,381						11,105,989					
Number of persons	65,802						750,390					65,118						735,700					

% refers to percentage of total risk time. Deaths refer to deaths from stroke. Rate is number of deaths per person years multiplied by 1,000.

Descriptive statistics for region of residence and calendar year are not displayed.

Of all sibling deaths, 4.9% were due to stroke and 15.5% were due to an external cause. Stroke refers to ICD 8 and ICD 9 codes 430–438, and ICD 10 codes I60–I69. External cause refers to ICD 8 codes E800–E999, ICD 9 codes E807–E999, and ICD 10 codes V01-Y98.

To adjust for characteristics differences between these two groups (see Table 1), we estimated standardized risk ratios of fatal stroke, where we adjusted for the effects of all the control variables. Estimates for the effects of the control variables were much in line with expectations (Table S1), and there was an association with a sibling’s death in most subgroups, albeit the statistical power was small (Table S2).

Also in the adjusted models (Table 2), the risk of fatal stroke was notably higher in persons who had experienced the death of sibling than in those who had not experienced the death of a sibling. In women, the risk ratio was 1.31 (95% CI: 1.05−1.62), whereas it in men was 1.11, but statistically not significant (95% CI: 0.92−1.33). Distinguishing between different main types of stroke revealed that the association was strongest for ischemic stroke, but since the number of deaths was small we could not gain any statistically significant estimates (Table S3).

**Table 2:** Standardized effect of sibling’s death on stroke mortality.

	Men		Women	
Cause of sibling’s death				
All causes	1.11	(0.92–1.33)	1.31	(1.05–1.62)
External	0.77	(0.51–1.17)	1.47	(1.00–2.17)
Not external	1.20	(0.99–1.46)	1.26	(1.00–1.60)
Stroke	1.78	(1.00–3.17)	1.44	(0.64–3.24)
Not stroke	1.08	(0.89–1.30)	1.30	(1.04–1.62)

Numbers are mortality risk ratios (with 95% confidence intervals) between exposed and unexposed index persons, i.e., the ratio of the death risk of persons with a deceased sibling and the death risk of persons with no deceased sibling, adjusted for effects of all control variables. Control variables included in the estimations are age, calendar year, socioeconomic status, marital status, number of children, number of siblings and region of residence. In the models where we distinguish between external and not external cause of sibling’s death, the former refers to ICD 8 codes E800–E999, ICD 9 codes E807–E999, and ICD 10 codes V01-Y98, and the latter to all other codes. In the models where we distinguish between stroke and not stroke as the cause of sibling’s death, the former refers to ICD 8 and ICD 9 codes 430–438, and ICD 10 codes I60–I69, and the latter to all other codes. All models have been estimated separately for men and women.

**Figure 1:**
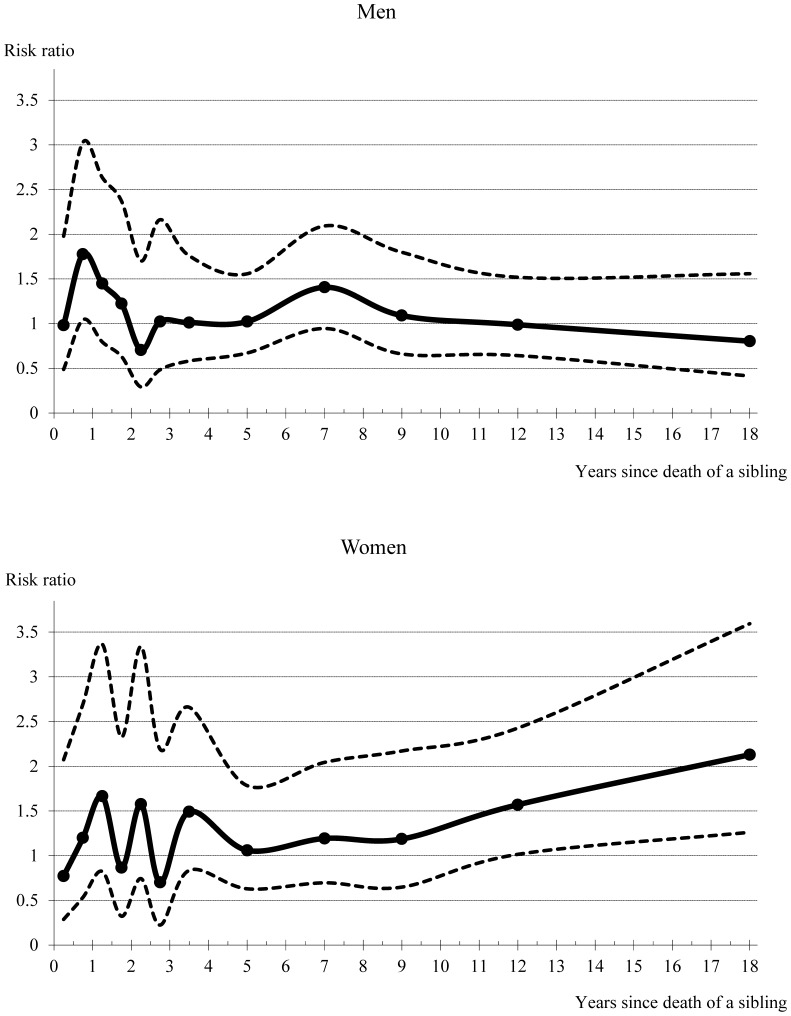
Standardized stroke mortality risk, with 95% confidence intervals, according to time since the death of a sibling (from any cause). Notes: Reference group is persons without sibling loss. The time intervals applied, which also fitted the data best, are Q1–Q2, Q3–Q4, Q5–Q6, Q7–Q8, Q9–Q10, Q11–Q12, Q13–Q16, Q17–Q24, Q25–Q32, Q33–Q40, Q41–Q60, and Q61–Q88. In the figure, the dots are set at the midpoint of each interval. In a simplified model that separates between a short-term effect (less than two years since the death of a sibling) and a long-term effect (at least two years since the death of a sibling), the estimate for the former was 1.37 (95% CI: 0.89−2.11), and the estimate for the latter 1.08 (95% CI: 0.89−1.31) in men. In women, the corresponding estimates were 0.98 (95% CI: 0.52−1.85), and 1.35 (95% CI: 1.08−1.68).

In both sexes, we observed a short-term increase in the stroke mortality risk, particularly around the first year subsequent to a sibling’s death (Figure 1). In men, there was an approximately 1.8 fold increase in stroke mortality risk one year after a sibling’s death. Also for women there seemed to be a short-term effect, but there was much more random variation and the point estimates were statistically not significant. In contrast to men, women appeared to suffer also from long-term effects of bereavement, as the stroke mortality risk showed a tendency to increase over longer follow-up periods.

More detailed analyses of the cause of sibling’s death revealed that at least part of the bereavement effect in men could be due to residual confounding. There was no increase in the risk of fatal stroke if the sibling died from an external cause (Table 2), whereas a person who experienced a sibling’s death from stroke had an own risk of fatal stroke that was 1.78 that of a person without sibling loss (95% CI: 1.00−3.17). By contrast, the stroke mortality of women who had experienced a sibling’s death from an external cause was 1.47 that of women without sibling loss (95% CI: 1.00−2.17), and the ratio was 1.30 (95% CI: 1.04−1.62) if the sibling had died from any other cause than stroke. This pattern suggests that, in women, the increased stroke mortality risk associated with a sibling’s death may not be purely a reflection of shared genetic risk factors.

## Discussion

This large-scale follow-up study based on the Swedish population register adds to the literature on bereavement as another potential trigger of stroke. We found that the death of a sibling was associated with an increased overall risk of fatal stroke, primarily in women. For both men and women, there was a short-term increase in stroke mortality around the first year after the death of a sibling whereas longer-term elevation in the risk was found primarily for women. Analyses of cause-specific deaths suggest that at least in women the association is not purely a reflection of shared genetic predisposition to stroke or shared environmental determinants.

Bereavement following sibling loss could influence stroke mortality in the shorter-term through psycho-physiological stress mechanisms [1], [2] and could therefore explain the short-term increase in risk of stroke. However, chronic stressors could also lead to pathophysiological changes and contribute to the association months and some years after bereavement [2]. Adverse coping responses (e.g. unhealthy lifestyle behaviours) could also potentially explain the raised risk of stroke mortality in the long term [2], [3]. As we had no biologic or genetic data or information on health behaviours in our cohort, further studies are needed to test these conjectures.

Overall, women were found to be more vulnerable to the death of a sibling than men. The risk of fatal stroke was raised by 30 per cent among women who lost a sibling from any cause, which might reflect the argument that women place more emphasis on close social ties than men do, particularly when it comes to parents and the family [29], [30], and they might consequently experience the loss as more stressful. If the sibling died from an external cause, the risk of fatal stroke in women was almost 50 per cent higher as compared with persons with no sibling loss. This may reflect an additional exposure to stress, attendant on the unfortunate circumstance of losing a sibling through an external cause such as an accident, suicide or homicide. The risk was 30 per cent higher if the cause of the sibling’s death was from any other cause besides stroke. The fact that the associations between sibling’s death and stroke mortality from discordant causes were of similar magnitude as the association when both siblings died of the same cause (both died of stroke) strengthens the possibility that the association may be causal among women. If we would have found an association only when both siblings died of stroke, confounding by genetic similarities or shared environmental conditions would seem more likely. Nevertheless, in order to exclude the possibility of confounding by genetic similarities and shared environmental conditions between siblings, information on biological and genetic data as well as shared childhood social conditions would be necessary. Such information was unfortunately not included in the registry data used in this study.

An association between sibling loss and fatal stroke was found in the short term among both men and women. Previous research has suggested that women are more vulnerable to psycho-physiological stress mechanisms such as the “broken heart syndrome” [26], [27] but also that men are more vulnerable to bereavement in the shorter-term [31]. In women, we also found indications of persistent excess mortality up to 12+ years after a sibling’s death. Hence, the risk period of bereavement seems to last longer among women than among men which is consistent with some previous research [25]. Maladaptive coping behaviours may emerge over a period of some years among women, leading to lagged effects of sibling loss on bereaved women’s risk of stroke. It is also plausible that women’s larger primary network (such as family and relatives) [29], [30] can help them cope with the grief in the immediate aftermath and postpone some of the bereavement effect.

Despite the obvious strengths of this study such as the use of total population register data, longitudinal follow-up, reliable information on stroke mortality and other included variables, some limitations should be noted. Despite the large population, the total number of stroke cases was low in people that experienced a sibling’s death (less than 300). This may have limited the power of this study. Moreover, more detailed individual information is required to uncover the actual causal mechanisms that link sibling deaths and stroke mortality as well as to reduce the risk of confounding. Such information could also minimize the possibility of omitted variable bias. Ideally, one would like to have access to biological and genetic data, detailed information on diseases from medical records, more information on shared childhood social environment and family characteristics and detailed data on personal and relational characteristics which is not included in the registers. On the other hand, our results likely underestimate the true bereavement effect on the risk of stroke, since we could study only fatal stroke. Examining incident stroke would provide more precision and greater statistical power for the estimates. The absence of a relationship between sibling loss and fatal stroke during the first few months of bereavement might be explained, for instance, by the fact that non-fatal stroke events were not analysed and stroke mortality does not necessarily coincide with the specific event of stroke, but with a lag.

Our findings suggest that the healthcare system should be concerned about broader collateral health effects of illness and death on the decedent’s social networks [32]. Our findings suggest that it is important for health care workers to monitor bereaved siblings shortly after the death of a sibling and recognize signs of acute or chronic psycho-social stress mechanisms that could lead to risk of stroke. Yet our findings conform also to the view that it could be important to follow bereaved siblings over time and recognize potential changes of health related behaviours that could lead to risk of stroke [33].

The study provides the first large-scale evidence for increased stroke mortality associated with the death of a sibling. The mechanisms linking the death of a sibling and fatal stroke events among the bereaved sibling, and women in particular, need to be further investigated.

## Supporting Information

Table S1 **Risk ratios of stroke mortality.**
(DOCX)

Table S2 **Standardized effect of sibling’s death (from any cause) on stroke mortality stratified by age category, socioeconomic status, marital status, number of children, and number of siblings.**
(DOCX)

Table S3 **Standardized effect of sibling’s death (from any cause) on different main types of fatal stroke.**
(DOCX)
