# Supplementary material for: Fatal Stroke after the Death of a Sibling: A Nationwide Follow-Up Study from Sweden
Source: PLoS One. 2013 Feb 22;8(2):e56994. doi: 10.1371/journal.pone.0056994 (PMC3579925; doi:10.1371/journal.pone.0056994)
Supplement: Table S1 — Risk ratios of stroke mortality. (DOCX) [file pone.0056994.s001.docx]

| Table S1. Risk ratios of stroke mortality | | |  |  |  |
| --- | --- | --- | --- | --- | --- |
|  | Men | |  | Women | |
|  |  |  |  |  |  |
|  |  |  |  |  |  |
| Age in years |  |  |  |  |  |
| 40-44 | 1 |  |  | 1 |  |
| 45-49 | 1.85 | (1.55-2.21) |  | 1.49 | (1.22-1.81) |
| 50-54 | 3.29 | (2.76-3.91) |  | 2.65 | (2.17-3.22) |
| 55-59 | 6.31 | (5.28-7.55) |  | 3.52 | (2.84-4.35) |
| 60-64 | 12.97 | (10.73-15.68) |  | 6.25 | (4.96-7.87) |
| 65-69 | 25.36 | (20.18-31.88) |  | 12.85 | (9.74-16.96) |
|  |  |  |  |  |  |
| Socioeconomic status |  |  |  |  |  |
| Blue-collar worker | 1 |  |  | 1 |  |
| White-collar worker | 0.69 | (0.62-0.77) |  | 0.73 | (0.64-0.84) |
| Self-employed | 0.91 | (0.78-1.05) |  | 0.93 | (0.71-1.22) |
| Outside labour market | 2.19 | (1.93-2.49) |  | 1.14 | (1.00-1.31) |
|  |  |  |  |  |  |
| Marital status |  |  |  |  |  |
| Married | 1 |  |  | 1 |  |
| Previously married | 1.64 | (1.44-1.88) |  | 1.69 | (1.45-1.96) |
| Never married | 1.45 | (1.26-1.67) |  | 1.35 | (1.14-1.61) |
|  |  |  |  |  |  |
| Number of children |  |  |  |  |  |
| 0 | 1 |  |  | 1 |  |
| 1 | 1.00 | (0.86-1.16) |  | 0.91 | (0.75-1.09) |
| 2 | 0.88 | (0.76-1.01) |  | 0.74 | (0.62-0.89) |
| >2 | 1.06 | (0.91-1.24) |  | 0.83 | (0.69-1.01) |
|  |  |  |  |  |  |
| Number of siblings |  |  |  |  |  |
| 1 | 1 |  |  | 1 |  |
| 2 | 1.04 | (0.93-1.16) |  | 1.17 | (1.02-1.34) |
| >2 | 1.06 | (0.96-1.18) |  | 1.16 | (1.01-1.32) |
| Numbers are mortality risk ratios (with 95% confidence intervals) | | | | | |
| from a model with main effects that includes also sibling’s death | | | | | |
| (from any cause), region of residence, and calendar year as | | | | | |
| covariates. |  |  |  |  |  |
| Separate models have been estimated for men and women. | | | | | |
