# Supplementary material for: Fatal Stroke after the Death of a Sibling: A Nationwide Follow-Up Study from Sweden
Source: PLoS One. 2013 Feb 22;8(2):e56994. doi: 10.1371/journal.pone.0056994 (PMC3579925; doi:10.1371/journal.pone.0056994)
Supplement: Table S2 — Standardized effect of sibling’s death (from any cause) on stroke mortality stratified by age category, socioeconomic status, marital status, number of children, and number of siblings. (DOCX) [file pone.0056994.s002.docx]

| Table S2. Standardized effect of sibling’s death (from any cause) on stroke mortality stratified by age category, socioeconomic status, marital status, number of children, and number of siblings | | | | | | |
| --- | --- | --- | --- | --- | --- | --- |
|  | Men | |  | Women | |  |
|  |  |  |  |  |  |  |
|  |  |  |  |  |  |  |
| Age in years |  |  |  |  |  |  |
| 40-44 | 1.15 | (0.54-2.46) | | 1.32 | (0.61-2.84) | |
| 45-49 | 1.17 | (0.70-1.96) | | 1.41 | (0.81-2.45) | |
| 50-54 | 1.08 | (0.73-1.62) | | 1.51 | (1.02-2.25) | |
| 55-59 | 0.81 | (0.56-1.18) | | 1.35 | (0.91-2.02) | |
| 60-64 | 1.21 | (0.89-1.65) | | 1.23 | (0.82-1.86) | |
| 65-69 | 1.44 | (0.97-2.15) | | 0.96 | (0.54-1.70) | |
|  |  |  |  |  |  |  |
| Socioeconomic status |  |  |  |  |  |  |
| Blue-collar worker | 1.13 | (0.88-1.45) | | 1.08 | (0.77-1.52) | |
| White-collar worker | 1.05 | (0.73-1.50) | | 1.24 | (0.84-1.84) | |
| Self-employed | 1.21 | (0.74-1.97) | | 1.99 | (0.94-4.23) | |
| Outside labour market | 1.10 | (0.77-1.57) | | 1.56 | (1.11-2.18) | |
|  |  |  |  |  |  |  |
| Marital status |  |  |  |  |  |  |
| Married | 1.07 | (0.84-1.37) | | 1.09 | (0.82-1.44) | |
| Previously married | 1.56 | (1.09-2.24) | | 1.10 | (0.69-1.74) | |
| Never married | 0.95 | (0.69-1.30) | | 2.27 | (1.59-3.26) | |
|  |  |  |  |  |  |  |
| Number of children |  |  |  |  |  |  |
| 0 | 1.21 | (0.89-1.64) | | 1.57 | (1.02-2.42) | |
| 1 | 0.76 | (0.49-1.20) | | 1.34 | (0.87-2.06) | |
| 2 | 1.29 | (0.96-1.73) | | 1.11 | (0.78-1.60) | |
| >2 | 1.06 | (0.76-1.48) | | 1.36 | (0.95-1.94) | |
|  |  |  |  |  |  |  |
| Number of siblings |  |  |  |  |  |  |
| 1 | 1.27 | (0.88-1.84) | | 1.29 | (0.80-2.06) | |
| 2 | 0.97 | (0.68-1.38) | | 1.06 | (0.70-1.61) | |
| >2 | 1.12 | (0.89-1.42) | | 1.43 | (1.09-1.87) | |
|  |  |  |  |  |  |  |
| Numbers are standardized mortality ratios (with 95% confidence | | | | | | |
| intervals) between exposed and unexposed index persons, i.e., | | | | | | |
| the ratio of the death risk of persons with a deceased sibling | | | | | |  |
| and the death risk of persons with no deceased sibling, adjusted | | | | | | |
| for effects of all control variables. | | |  |  |  |  |
| Control variables included in the estimations are age, calendar | | | | | |  |
| year, socioeconomic status, marital status, number of children, | | | | | | |
| number of siblings, and region of residence. | | | |  |  |  |
| The results are based on five different specifications for each | | | | | |  |
| sex, where we in each model with all main effects have | | | | | |  |
| included also the joint effect of sibling’s death and the control | | | | | | |
| variable of interest. |  |  |  |  |  |  |
| All models have been estimated separately for men and women. | | | | | | |
