# Supplementary material for: Fatal Stroke after the Death of a Sibling: A Nationwide Follow-Up Study from Sweden
Source: PLoS One. 2013 Feb 22;8(2):e56994. doi: 10.1371/journal.pone.0056994 (PMC3579925; doi:10.1371/journal.pone.0056994)
Supplement: Table S3 — Standardized effect of sibling’s death (from any cause) on different main types of fatal stroke. (DOCX) [file pone.0056994.s003.docx]

| Table S3. Standardized effect of sibling’s death (from any cause) on different  main types of fatal stroke | | | | | | |
| --- | --- | --- | --- | --- | --- | --- |
|  | Men | |  | Women | |  |
|  |  |  |  |  |  |  |
|  |  |  |  |  |  |  |
| Stroke type |  |  |  |  |  |  |
|  |  |  |  |  |  |  |
| Subarachnoid | 1.12 | (0.69-1.81) | | 1.35 | (0.94-1.94) | |
|  |  |  |  |  |  |  |
| Hemorrhagic | 1.08 | (0.87-1.34) | | 1.22 | (0.91-1.63) | |
|  |  |  |  |  |  |  |
| Ischemic | 1.26 | (0.74-2.17) | | 1.69 | (0.78-3.66) | |
|  |  |  |  |  |  |  |
| Numbers are mortality risk ratios (with 95% confidence | | | | | |  |
| intervals) between exposed and unexposed index persons, i.e., | | | | | | |
| the ratio of the death risk of persons with a deceased sibling | | | | | | |
| and the death risk of persons with no deceased sibling, | | | | | |  |
| adjusted for effects of all control variables. | | | | |  |  |
| Control variables included in the estimations are age, calendar | | | | | | |
| year, socioeconomic status, marital status, number of children, | | | | | | |
| number of siblings, and region of residence. | | | | |  |  |
| Subarachnoid refers to ICD 8 and ICD 9 codes 430, and | | | | | |  |
| ICD 10 codes I60. Hemorrhagic refers to ICD 8 and ICD 9 | | | | | |  |
| codes 431-434, and ICD 10 codes I61-I66. Ischemic refers to | | | | | | |
| ICD 8 and ICD 9 codes 435-438, and ICD 10 codes I67-I69. | | | | | | |
| Subarachnoid accounts for 28.1% of all deaths from stroke, | | | | | |  |
| hemorrhagic for 63.5%, and ischemic for 8.4%. | | | | | |  |
| All models have been estimated separately for men and women. | | | | | | |
